# Complete mitochondrial genome of the Japanese Cormorant *Phalacrocorax capillatus* (Temminck & Schlegel, 1850) (Suliformes: Phalacrocoracidae)

**DOI:** 10.1080/23802359.2022.2113753

**Published:** 2022-08-30

**Authors:** Rina Honda, Mizue Inumaru, Yukita Sato, Atsushi Sogabe

**Affiliations:** a Saitama Museum of Natural History, Nagatoro, Japan; b Department of Medical Entomology, National Institute of Infectious Diseases, Tokyo, Japan; c Department of Veterinary Medicine, Nihon University, Fujisawa, Japan; d Department of Biology, Hirosaki University, Hirosaki, Japan

**Keywords:** *Phalacrocorax capillatus*, mitogenome, phylogenetic tree

## Abstract

The complete sequencing of mitochondrial DNA of the Japanese Cormorant *Phalacrocorax capillatus* was performed using long PCR and primer walking methods. The assembled genome was 19,105 bp in length. It contained 13 protein-coding genes, two ribosomal RNA genes, 22 transfer RNA genes, and two control regions. The phylogenetic analysis using the obtained sequence showed that *P. capillatus* is closest to *P. carbo*.

The Japanese Cormorant *Phalacrocorax capillatus* (Temminck & Schlegel, 1850) is native to Northeast Asia, from Taiwan, throughout the east coast of China, Korea, and Japan to Sakhalin (Orta et al. 2020). The phylogenetic position of *P. capillatus* in the family Phalacrocoracidae remains controversial as it forms a monophyletic group with some subspecies of *P. carbo* (Kennedy and Spencer 2014). This ambiguity in the phylogenetic position of *P. capillatus* is possibly be due to lack of genetic data. Therefore, the development of genetic markers for *P. capillatus* with varying evolutionary rates is needed to understand the evolution of Phalacrocoracidae.

A blood specimen of *P. capillatus* was obtained from a bird rescued in Aomori City, Japan (40°48′ N, 140°46′ E) on 1 February 2010, by the Aomori Wildlife Conservation Center; however, the bird died two days later. The genomic DNA extracted from the specimen was deposited at Hirosaki University (Dr. Atsushi Sogabe, e-mail: atsushi.sogabe@hirosaki-u.ac.jp) under voucher number HUA2103161. The complete mitogenome sequence was determined using primer walking for the five long PCR products (see Table S1 for a list of primers used for long PCR). The assembled mitogenome sequence was annotated using MITOS web server (Bernt et al. 2013).

The complete mitogenome of *P. capillatus* was 19,105 bp in length (DDBJ accession no. LC714913). It contained 13 protein-coding genes (PCGs), two rRNA genes, 22 tRNA genes, and two control regions. The gene arrangement of *P. capillatus* was identical to that of other Suliformes species, characterized by a duplicated region spanning from the latter half of *cytochrome b* to the control region (Gibb et al. 2013). Most mitochondrial genes are encoded on the H-strand, except for *ND6* and eight tRNA genes (*tRNA-Gln*, *-Ala*, *-Asn*, *- Cys*, *-Tyr*, *-Ser*, *-Glu*, and *-Pro*). The overall nucleotide composition was as follows: A (31.9%), C (31.9%), G (13.2%), and T (22.9%).

The maximum-likelihood method was used to reconstruct the phylogenetic tree based on 13 PCGs from 11 species of Suliformes, with Grey Heron *Ardea cinerea* (Pelecaniformes) as an outgroup. The best-fitting model of sequence evolution was selected using ModelTest-NG 0.1.6 (Darriba et al. 2020). Phylogenetic analysis was conducted using RAxML-NG 1.0.1 (Kozlov et al 2019) with 1000 pseudoreplicates to estimate branch support values. The overall topology among the families of Suliformes was congruent with that of Gibb et al. (2013); however, the phylogenetic relationships within the family Phalacrocoracidae differed from those of Kennedy and Spencer (2014) (Figure 1). We also found that *P. capillatus* is a sister species of *P. carbo*. The present study provides useful genetic tools to facilitate further studies on the Phalacrocoracidae evolution, as well as the population genetics of the Japanese Cormorant.

**Figure 1.**
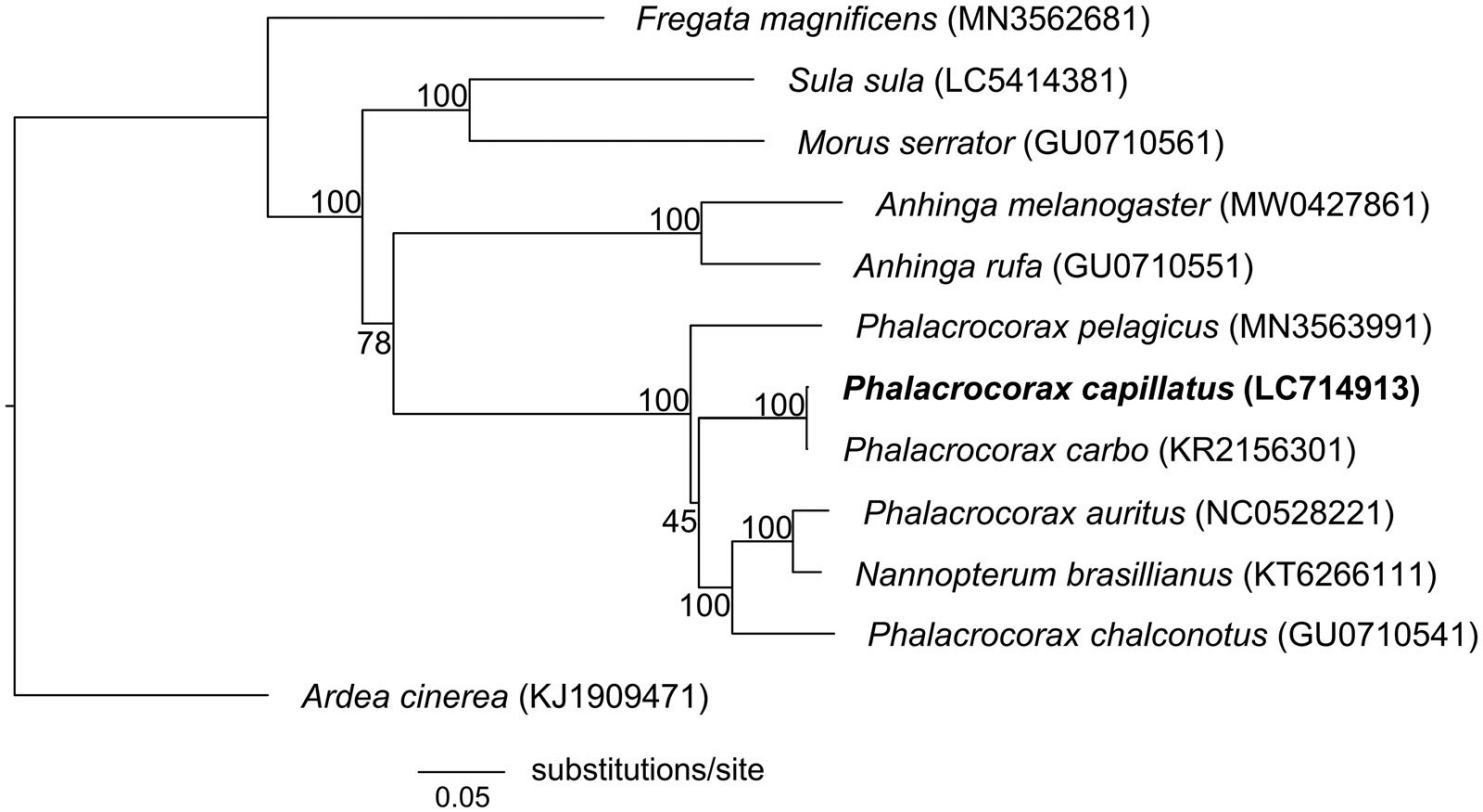
Maximum-likelihood tree of the order Suliformes based on the sequences of concatenated 13 PCGs with Grey Heron *Ardea cinerea* as an outgroup. Numbers beside each node indicate bootstrap support values.

## Supplementary Material

Supplemental Material

## Data Availability

The data that support the findings of this study are available in DDBJ at https://www.ddbj.nig.ac.jp/index-e.html (reference number: LC714913).
