# Supplementary material for: Complete mitochondrial genome of the Japanese Cormorant Phalacrocorax capillatus (Temminck & Schlegel, 1850) (Suliformes: Phalacrocoracidae)
Source: Mitochondrial DNA B Resour. 2022 Aug 30;7(8):1577–8. doi: 10.1080/23802359.2022.2113753 (PMC9448395; doi:10.1080/23802359.2022.2113753)
Supplement: Supplemental Material [file TMDN_A_2113753_SM9599.pdf]

Table S1 Primers used for the long PCR of mitochondrial DNA in *Phalacrocorax capillatus*. Primer3* was used to develop the primers, using the mitogenome of *Phalacrocorax carbo* (Genbank accession No. KR21563) as the reference sequence.

Primer name		Region / position (5’)	Primer Sequence	Size (bp)
PC_12S_COI	F	12S rRNA / 702	5’- AAACAGCCTACATACCGCCG -3’	5186
	R	COI / 5887	5’- GATTGCCCCCAGGATTGAGG -3’	
PC_COI_ATP6	F	COI / 5566	5’- TGTAATTGTCACCGCCCACG -3’	2935
	R	ATP6 / 8500	5’- GTGAGGTTTGCTGTGAGTCG -3’	
PC_ATP6_Cytb (0)	F	ATP6 / 8358	5’- CGAAACCAACCAACCATCTC -3’	5897
	R	CYTB / 14254	5’- TGTGGTGAGACCTGCGATTA -3’	
PC_Cytb (0)_D-loop (0)	F	CYTB / 13926	5’- ATGGCGCATCATTCTTCTTC -3’	2597
	R	D-loop / 16522	5’- TGGGTGGGTAGTGAAATTGG -3’	
PC_D-loop (0)_12S	F	D-loop / 16192	5’- GGCCTCTCCTCTTTTGGTTC -3’	3775
	R	12S rRNA / 861	5’- TCACGTCCCCTTTCGTTAAG -3’	

* Untergasser A, Cutcutache I, Koressaar T, Ye J, Faircloth BC, Remm M, Rozen SG (2012) Primer3 -new capabilities and interfaces. Nucleic Acids Research 40 (1): e115.
